# Temperamental and Personality Traits as Factors Related to Changes in Health Behaviors and Quality of Life in Patients With Metabolic Syndrome in Poland

**DOI:** 10.3389/fpsyg.2021.709935

**Published:** 2021-09-10

**Authors:** Agnieszka Burnos, Andrzej Skrobowski

**Affiliations:** ^1^Psychology Department, The University of Warsaw, Warsaw, Poland; ^2^Warsaw Military Institute, Warsaw, Poland

**Keywords:** metabolic syndrome, personality traits, temperamental traits, quality of life, motivational intervention

## Abstract

Lifestyle changes in diet and physical activity are necessary for managing metabolic syndrome. The aim of this longitudinal study was to examine temperamental and personality traits as moderators of lifestyle changes prompted by motivational intervention. The sample consisted of 50 patients aged 22–65years (*M*=45.26; *SD*=9.79) who fulfilled the diagnostic criteria for metabolic syndrome and were undergoing treatment at the Military Institute of Medicine in Warsaw. There were two measurements: an initial measurement and a second 15months after motivational counseling. Each patient completed the questionnaires: Formal Characteristics of Behavior – Temperament Inventory, NEO Five Factor Inventory, Inventory of Health Behavior, and Short Form Survey SF-36. Body Mass Index (BMI), Fat Mass, Fat-free Mass, Intracellular Water, and Basal Metabolic Rate (BMR) were also measured. Data were analyzed using dependent samples *t*-tests to detect the changes in consecutive measurements, the hierarchical regression analysis was used to investigate temperamental and personality traits as predictors of change, the cluster analysis was used to extract the subgroups of patients with distinct profiles of temperamental and personality traits, and the analysis of variance was used to analyze extracted profiles as potential moderators of change. Three subgroups were extracted using k-means clustering: patients with higher Neuroticism, Perseveration, and Emotional Reactivity; patients higher Extraversion, Briskness, Sensory Sensitivity, Endurance, Activity, and Conscientiousness; and patients with lower Perseveration. All patients improved significantly in terms of physical quality of life (QoL), health behaviors, BMI, BMR, and Fat-free Mass (*p*<0.05). Regression analysis found that higher Sensory Sensitivity, lower Perseveration, and higher Agreeableness fostered positive change (*p*<0.05). Patients with higher Neuroticism, Perseveration, and Emotional Reactivity also improved in terms of their Emotional Quality of Life and Health Practices, reaching parity with other patients, which was verified on the basis of statistically significant interaction (*p*<0.05). The temperamental and personality trait profiles moderated the changes in health practices and emotional QoL. Motivational counseling was effective for patients diagnosed with metabolic syndrome in general, but patients with higher Neuroticism, Perseveration, and Emotional Reactivity benefited even more, as they were in poorer psychological condition before the motivational intervention.

## Introduction

Metabolic syndrome is a pathological state that is characterized by abdominal obesity, insulin resistance, hypertension, and hyperlipidemia (Saklayen, 2018). It increases the risk of chronic diseases like type 2 diabetes and coronary diseases and also increases the probability of stroke and other disabilities. It is estimated that metabolic syndrome affects about 25% of the world population – over a billion people. Metabolic syndrome in Poland is present in about 20% of the adult population – 5.8 million people (Kalinowski and Mianowana, 2016). According to the definition of the World Health Organization (Alberti and Zimmet, 1998), metabolic syndrome is constituted by insulin resistance or glucose >6.1mmol/L (110mg/dl), 2h glucose >7.8mmol (140mg/dl), and at least two of the following: HDL cholesterol <0.9mmol/L (35mg/dl) in men, < 1.0mmol/L (40mg/dl) in women; triglycerides >1.7mmol/L (150mg/dl); waist/hip ratio > 0.9 (men) or > 0.85 (women) or Body Mass Index (BMI)>30kg/m^2^; blood pressure > 140/90mmHg.

The pathophysiological mechanisms of metabolic syndrome have been studied, and the consumption of high calorie/low fiber fast food as well as low levels of physical activity are indicated as the main causes of metabolic syndrome (Barrès and Zierath, 2016; Bird and Hawley, 2017). Genetic predispositions have also been studied; however, they were found to be only a minor factor (Locke et al., 2015). Guidelines for people diagnosed with metabolic syndrome and for people at risk of developing it in terms of physical activity and appropriate diet have been formulated at the individual level (de la Iglesia et al., 2014; Salas-Salvadó et al., 2014; Clark et al., 2019) and in terms of general health policy (Peeters and Backholer, 2017). However, there is also a need for motivational interventions to implement and maintain these guidelines. For decades, the literature has emphasized the necessity of recognizing individual needs, goals, and choices as well as the importance of feedback and reward systems as an important basis for solid motivation (Oldridge and Stoedefalke, 1984). An individual approach to motivational training improves patients’ participation and strengthens their ability to make changes to their lifestyle. Self-efficacy is one of the most important factors in predicting failures and successes in maintaining motivation (Dohnke et al., 2010). A systematic review of 14 studies concluded that motivational interventions are effective at fostering long-term behavioral changes and improvement of self-efficacy (McGrane et al., 2015). It was found that self-regulatory strategies indirectly influence physical activity through self-efficacy in patients diagnosed with diabetes or metabolic syndrome (Olson et al., 2017). Self-efficacy in regulating eating habits was one of the predictors of adherence in a prevention program for patients with metabolic syndrome (Susin et al., 2016). A systematic review and meta-analysis (Kuo et al., 2014) point out that the empowerment-based self-management interventions for patients with chronic metabolic diseases, including metabolic syndrome, were effective at increasing self-efficacy.

However, there are still individual differences in patients’ adherence to explain. To do so, personality and temperamental traits have been investigated as possible predictors of compliance with health guidelines and as predictors of eating behaviors and physical activity in general (Walther and Hilbert, 2016; Reshadat et al., 2017; Stanyte and Smigelskas, 2018). Personality traits have also been examined as predictors of metabolic syndrome (Sutin et al., 2010). High Neuroticism, low Agreeableness, and low Conscientiousness have been found to be associated with metabolic syndrome. Weak associations have been found between energy expenditure at peak walking pace and Neuroticism, Extraversion, Openness, and Conscientiousness (Terraciano et al., 2013). However, one systematic review points out that there is no clear association between personality and metabolic syndrome – either its occurrence or its development (Mommersteeg and Pouwer, 2012).

Strelau’s Regulative Theory of Temperament (RTT; Strelau, 1996) describes formal aspects of behavior, including energetic and temporal characteristics composed of specific traits such as Sensory Sensitivity, Emotional Reactivity, Endurance and Activity (energetic aspect), Briskness and Perseveration (temporal characteristics). Sensory Sensitivity characterizes one’s capacity to perceive weak sensory stimuli. Emotional reactivity is the tendency to respond with intensity to stimuli, which induce emotions. Endurance is the ability to withstand long-lasting or exhausting conditions. Activity is the inclination to engage in behaviors that take place under intensely stimulating conditions. Briskness is the ability to react quickly and shift from one behavior to another. Perseveration is the tendency to repeat emotional states in reaction to stimuli, even if the stimuli are no longer present. Many research projects have investigated the functional significance of the temperamental characteristics postulated by the RTT. These temperamental traits may act as the moderators of the impact of life events by increasing or decreasing the associated stimulation (Strelau, 2006). They may also affect coping strategies used in stressful situations. Specifically, they can affect the regulation of emotional states. Temperament is distinguished from character, which is defined as the self-regulatory aspect of personality – the way people shape and adapt responses to changing external and internal conditions (Cloninger et al., 2019). Temperamental traits postulated in the regulative theory of temperament may also be considered as endophenotypes for obesity (Oniszczenko et al., 2015).

The five-factor approach (McCrae and Costa, 1999) is the most popular and influential way of describing human personality. It describes personality in terms of five personality traits: Neuroticism, Extraversion, Openness to Experience, Agreeableness, and Conscientiousness. They do not only describe the energetic and temporal aspects of individual differences in human behavior but also include attitudes and reactions to social events. Neuroticism is a tendency to be emotionally unstable and to experience negative emotions due to low tolerance for stress. Extraversion is characterized by engagement in external activities, usually with other people. Openness to Experience is characterized by intellectual curiosity and appreciation of adventure and imagination. Agreeableness is a general concern for getting along with others and being kind, helpful, and trustworthy. Conscientiousness is a trait constituted by self-discipline, reliability, and a preference for planning rather than spontaneity. The use of both temperamental and personality traits allows for a more accurate representation of individual differences in patients’ compliance.

The role of personality traits as predictors of eating habits has been examined in the general population and in specific samples. One systematic review has found that neuroticism, impulsivity, and sensitivity to reward are risk factors for obesity (Gerlach et al., 2015), while conscientiousness and self-control have a protective function against possible weight gain. Specific dimensions of personality can interact with each other. Conscientiousness is the basis for the regulation of internal urges and a foundation of self-discipline. It may function as a source of control over compulsive eating behaviors, which may be strengthened by neuroticism. A link between lower conscientiousness and obesity has also been found in children (Sutin and Terracciano, 2018).

Patients with obesity have not only high scores on neuroticism but also have low scores for Agreeableness, Extraversion, Conscientiousness, and Openness to Experience (Garrido et al., 2018). The social attitude characteristic of Extraversion, on the other hand, has been found to foster improvement in multimodal obesity treatment (Lahmann et al., 2011). However, the associations between social activity and anthropometric outcomes were more clear in women than in men (Hosseini et al., 2020).

The positive correlation between bipolar spectrum features and neuroticism, as well as the negative correlation of such features with Agreeableness and Conscientiousness, has been examined in the context of obesity (Dudek et al., 2015). The results suggest that it is the ability to exercise control over constant or situational emotional tension that is crucial for understanding one’s eating behaviors. Personality traits, situations, or a combination of both can generate emotional tension, which necessitates a higher level of self-control to protect an individual from engaging in compulsive eating. The primary goal of compulsive eating is to release psychological tension, but an inevitable side effect is serious weight gain. However, a propitious profile of personality traits can also serve as a basis for healthy behaviors. It was found in a large sample of 5,150 adult participants that lower neuroticism and higher Conscientiousness were not only related to lower BMI but also that participants with such a profile of personality traits tend to engage in physical activity and pay attention to their diet in terms of ingredients and regular meal rhythms (Sutin and Terracciano, 2016).

In order to understand health behaviors, a broad approach is needed. The approach of Gochman (1988) takes into consideration convictions, expectations, thought patterns, and emotions. It allows for thinking about behaviors that increase or decrease the probability of developing a disease, behaviors engaged with conscious intention to maintain one’s current health status or to reduce the danger caused by disease, and behaviors that follow doctors’ recommendations. In this approach, health behaviors lead to a better quality of life (QoL). Based on the definition of The World Health Organization Quality of Life assessment (WHOQOL) (1995), QoL includes one’s evaluation of one’s physical health, emotional state, independence, and relations with one’s social environment. A chronic pathological state – such as metabolic syndrome, excessive weight, or obesity – changes one’s functioning in all these areas and one’s ability to improve the situation may depend on many factors. Temperamental and personality traits may be such factors.

The purpose of the current study is to analyze the role of temperamental and personality traits as moderators of change in health behaviors, in biological indicators of eating behaviors and in QoL in patients diagnosed with metabolic syndrome. The purpose of our research is to identify the profiles of temperamental and personality traits, which can facilitate positive change in health behaviors and, as a consequence, QoL as well as profiles that can impede such change in the context of metabolic syndrome. Our research question is whether temperamental and personality traits moderate such change and, on this basis, we formulated two hypotheses.

*H1:* There will be statistically significant changes in health behaviors, biological indicators, and QoL between the two consecutive measurements.

*H2:* Temperamental and personality traits of patients diagnosed with metabolic syndrome will be related to the magnitude of changes in health behaviors, biological indicators, and QoL between the two consecutive measurements.

From a practical point of view, if we find that person’s temperamental and personality traits profile matters, the measurement thereof should be incorporated into daily clinical practice with individual patients diagnosed with metabolic syndrome in order to predict and deal with patients’ potential strengths and difficulties when changing their health behaviors.

## Materials and Methods

This study was conducted at the Military Institute of Medicine in Warsaw, Poland. It is a military hospital; however, it is open to the general population. A total of 80 patients diagnosed with metabolic syndrome were recruited for the study; there were no inclusion criteria other than fulfilling the diagnostic criteria for the diagnosis. All patients that were diagnosed with metabolic syndrome for 12 consecutive months were asked by the attending doctor to participate. Patients younger than 18 and older than 65years old were excluded. Other exclusion criteria were chronic ischemic heart disease, diabetes being treated, confirmed chronic heart failure, confirmed chronic renal failure, cardiomyopathy, neoplastic disease, BMI higher than 45, pregnancy and diagnosed mental illness. The research design was longitudinal and consisted of two consecutive measurements. The period between the first and the second measurements was 15months. It was assumed that the length of this period was enough to detect long-lasting effects. From the initial sample of 80 patients, 50 (62.5%) participated in both measurements and these formed the final sample. All participants gave written consent for their data to be used in the research. The research design was approved by the Local Ethics Committee: the Bioethical Committee of the Military Institute of Medicine, decision no. 63/WIM/2011.

Indicators of metabolic syndrome, such as insulin resistance, HDL cholesterol, triglycerides, and blood pressure, were measured in a diagnostic process prior to participation in the study, before the first measurement. At the first measurement, the patients filled out four psychological questionnaires and were examined to determine five biological indicators: BMI, Fat Mass, Fat-free Mass, Intracellular Water, and Basal Metabolic Rate (BMR). A dietician measured the participants’ weight and height. Bioelectrical impedance analysis was used to measure Fat Mass, Fat-free Mass, and Intracellular Water. The psychological questionnaires were used to measure the temperamental and personality traits profiles of each patient as well as their health behaviors and QoL.

Temperament traits were assessed with the Formal Characteristics of behavior – Temperament Inventory (FCB-TI; Strelau and Zawadzki, 1995). This questionnaire measures six temperament traits: Briskness, Perseveration, Sensory Sensitivity, Emotional Reactivity, Endurance, and Activity. It has 120 items, with 20 items per scale (each scale can yield a total score of 0–20). The FCB-TI has good psychometric parameters and proven reliability and validity. Cronbach’s *α* varied from 0.72 to 0.86 depending on the scale.

Personality traits were measured with the use of the NEO Five Factor (NEO-FFI) Inventory, which consists of 60 items and measures Neuroticism, Extraversion, Openness to Experience, Agreeableness, and Conscientiousness – the dimensions of the five-factor approach described in the introduction. The questionnaire was also tested by the authors of its Polish adaptation (Zawadzki et al., 1998) to verify its reliability and validity. Cronbach’s *α* was in the range from 0.68 to 0.82 depending on the scale.

Health behaviors were assessed with the Inventory of Health Behavior (IHB; Juczyński, 2012). This inventory measures Correct Eating Habits, Preventive Behavior, Health Practices, and Positive Mental Attitude. Correct Eating Habits concern the type of food one eats. Preventive Behaviors refer to adherence to doctors’ advice and treatment. Health Practices refers to daily habits regarding rhythms of sleep and wakefulness as well as physical activity. Positive Mental Attitude refers to avoiding stressful events and situations evoking strong emotions. The inventory was developed in Poland and is widely used in the field of health psychology. Cronbach’s α was reported to be in the range from 0.60 to 0.65 depending on the scale.

Quality of life was measured with the use of the Polish version of the 36-item Short Form Survey (SF-36; Tylka and Piotrowicz, 2009). It consists of 36 questions about health and reactions to disease and measures two main dimensions of QoL. Physical Quality of Life refers to the physical sphere: from physical activity to pain and its negative consequences on daily activities. Emotional Quality of Life covers social activity, emotional consequences of restrictions resulting from a health condition, level of energy, and tiredness. In the Polish version, higher scores mean lower QoL and lower scores mean higher QoL. The inventory has good reliability and is commonly used in health economics to determine the cost-effectiveness of health treatments. Cronbach’s *α* was reported to be in the range from 0.75 to 0.95.

During the first session, in addition to the measurement with the questionnaires (FCB-TI, NEO-FFI, IHB, and SF-36) and the examination (including weight and height measurement and bioelectrical impedance analysis), participants were provided with diet recommendations and underwent motivational training (Miller and Rose, 2009). Information about diet recommendations was prepared and provided by a dietician. Patients were advised to avoid the consumption of saturated fat, trans-fatty acids, and cholesterol and were provided with prepared examples of daily meals. The motivational training was conducted by a health psychologist. During the training, patients were asked about their eating habits: their number of meals per day, eating between meals, number of sweets and snacks eaten, and their tendency to eat at night. The patients were also asked about their physical activity: what kinds of physical activity they undertook per month and per week. In the next stage, psychoeducation about the importance of a healthy diet and physical activity in metabolic syndrome was conducted. The long-term health consequences of metabolic syndrome were underlined. Also, the patients’ ambivalence and uncertainty about making major changes to their ways of life were discussed and the discrepancies between the patients’ values and goals and their health behaviors were elaborated upon. Possible changes – in the immediate term, in 1month, and over half a year – in each patient’s eating habits and physical activity were determined. All the interventions described above needed to be performed for each patient individually. Each meeting lasted for 50min.

During the second measurement, patients filled out the IHB and SF-36. Temperamental and personality traits were measured only once, during the first measurement, because psychological traits remain stable even over decades. Although they might change over a lifetime, 15months is too short to detect any differences. Health behaviors and QoL were measured twice. The same biological indicators were used in both measurements.

### Statistical Analysis

Because the distributions of the analyzed variables did not differ from the normal distribution (based on the skewness and kurtosis values), parametric statistical methods were used. Firstly, descriptive statistics were computed. The next stage was the analysis of differences between the two consecutive measurements. Their statistical significances were investigated with the use of the dependent samples *t*-test. Regression analysis was performed in order to verify the relationships between temperamental and personality traits and the magnitude of changes in health indicators and QoL. We wanted to analyze not only the possible effects of individual psychological traits but also the potential effects of configurations of temperamental and personality traits present in the sample. To this end, we first extracted profiles of patients in terms of temperamental and personality traits with k-means cluster analysis and then assessed the interactions between cluster membership and differences between the two consecutive measurements with mixed-model repeated-measures analysis of variance. Statistically significant interactions were interpreted with the use of 95% confidence intervals. With this sample size, an interaction effect will be statistically significant if Cohen’s *f* is equal to or greater than 0.29, allowing us to detect interactions that explain 7.5% of dependent variable variance. The power analysis was performed with the G*Power 3.1.9.2 software. All other calculations were made using IBM SPSS 25.0 software. The statistical significance level was equal to 0.05.

## Results

### Socio-Demographic Characteristics

Table 1 presents the socio-demographic characteristics of the initial sample and the studied sample. Most patients were married, had at least secondary education and were employed. The majority were males and lived in major cities with over 100, 000 inhabitants. The socio-demographic structure of the final sample was similar to the structure of the initial sample.

**Table 1:** Socio-demographic characteristics of the group of patients recruited for the study (*N*=80) and the group of patients participating in two measurements (*N*=50).

Variable	Patients recruited *N* =80 (%)	Two measurements *N* =50 (%)
**Gender**		
Female	15 (18.8%)	11 (22.0%)
Male	65 (81.2%)	39 (78.0%)
Age in years (*M*±*SD*)	46.15±9.84	45.26±9.79
**Marital Status**		
Married	70 (87.5%)	45 (90.0%)
Single	10 (12.5%)	5 (10.0%)
**Education**		
Elementary	3 (3.8%)	2 (4.0%)
Secondary	40 (50.0%)	24 (48.0%)
Higher Education	37 (46.2%)	24 (48.0%)
**Professional status**		
Employed	70 (87.5%)	46 (92.0%)
Retired	6 (7.5%)	3 (6.0%)
Unemployed	4 (5.0%)	1 (2.0%)
**Place of residence**		
Major city, over 100,000 inhabitants	55 (68.8%)	33 (66.0%)
Minor city, up to 100,000 inhabitants	20 (25.0%)	13 (26.0%)
Village	5 (6.2%)	4 (8.0%)

*M, Mean; SD, Standard Deviation*.

Out of 50 patients, 33 patients (66.0%) had BMI scores in the range between 25 and 30, indicating that they were overweight. A total of 17 patients (34.0%) had BMI scores higher than or equal to 30, which indicates obesity.

### Change Between Consecutive Measurements

Table 2 presents descriptive statistics for the analyzed variables acquired at the first and the second measurements. In the case of variables that were measured twice, dependent samples *t*-tests were computed. There were statistically significant changes in Physical Quality of Life (*p*<0.001), Correct Eating Habits (*p*<0.001), Preventive Behavior (*p*=0.004), Health Practices (*p*=0.042), BMI index (*p*=0.019), Fat-free Mass (*p*=0.046), and BMR (*p=0*.019). The mean value of the Physical Quality of Life was lower at the second measurement. Note, however, that the SF-36 inventory uses reverse scoring, meaning that lower values indicate better QoL. Within 15months, the patients acquired better Eating Habits, Preventive Behavior, Health Practices, lower BMI, higher Fat-free Mass, and lower BMR. These results reveal significant improvement in lifestyles and Physical Quality of Life. However, there were no statistically significant changes regarding Emotional Quality of Life (*p*=0.294), Positive Mental Attitude (*p*=0.249), Fat Mass (*p*=0.058), or Intracellular Water (*p*=0.451).

**Table 2:** Descriptive statistics for analyzed variables acquired in the first and second measurements with the values of statistical tests for differences.

Variables	First Measurement				Second Measurement				*t*	Value of *p*
	*M*	*SD*	*S*	*K*	*M*	*SD*	*S*	*K*		
Briskness	15.44	3.30	−0.79	0.24	–	–	–	–	–	–
Perseveration	11.30	4.20	−0.27	−0.07	–	–	–	–	–	–
Sensory Sensitivity	14.70	3.35	−0.62	−0.22	–	–	–	–	–	–
Emotional Reactivity	10.18	4.89	−0.23	−0.77	–	–	–	–	–	–
Endurance	9.36	4.64	−0.05	−0.37	–	–	–	–	–	–
Activity	8.04	4.55	0.15	−0.89	–	–	–	–	–	–
Neuroticism	86.45	20.24	0.40	−0.81	–	–	–	–	–	–
Extraversion	101.70	19.22	−0.26	0.91	–	–	–	–	–	–
Openness to Experience	101.32	17.85	0.80	0.52	–	–	–	–	–	–
Agreeableness	115.91	13.93	0.04	0.86	–	–	–	–	–	–
Conscientiousness	121.64	13.23	−0.67	0.63	–	–	–	–	–	–
Physical quality of life (QoL)	31.05	13.82	0.57	0.56	22.56	10.53	0.84	0.88	4.60	0.001
Emotional QoL	20.48	10.10	0.80	0.15	19.36	10.29	0.43	0.48	1.06	0.294
Correct Eating Habits	3.11	0.79	−0.26	−0.20	3.70	0.73	−0.85	0.87	−6.47	0.001
Preventive Behavior	3.38	0.65	−0.46	−0.52	3.64	0.61	−0.69	−0.28	−3.00	0.004
Health Practices	3.33	0.52	−0.03	0.51	3.56	0.59	−0.28	0.26	−2.09	0.042
Positive Mental Attitude	3.57	0.57	−0.97	0.38	3.69	0.50	−0.23	−0.80	−1.17	0.249
BMI Index	32.71	4.28	0.20	−0.03	32.25	3.85	0.83	−0.27	2.42	0.019
Fat Mass	31.76	9.53	0.46	0.24	30.34	8.47	0.74	0.66	1.94	0.058
Fat-free Mass	67.76	11.02	−0.36	−0.66	68.77	11.64	−0.50	−0.34	2.05	0.046
Intracellular Water	50.31	8.56	−0.36	−0.69	49.89	7.39	−0.40	−0.06	0.76	0.451
Basal Metabolic Rate	2060.32	349.67	−0.18	−0.67	2020.37	321.93	−0.23	−0.29	2.42	0.019

*Temperamental traits and personality traits were measured only once. M, mean value; SD, standard deviation; S, skewness; K, kurtosis; t, dependent samples t test; p, statistical significance*.

### The Role of Temperamental and Personality Traits

The first step in exploring the role of temperamental and personality traits was a set of hierarchical regression analyses. Each indicator of health, health behavior, and QoL that changed significantly from the first to the second measurement was analyzed in a separate model. The values from the second measurement were analyzed as dependent variables. The values of each indicator from the first measurement were included in each model in the first block with the use of the entry method, letting us to interpret the results of the regression analyses in terms of a difference between the first and the second measurements. The second block was concerned with temperamental traits and the third block with personality traits. Both temperamental traits and personality traits were entered stepwise into the model. The use of the stepwise method helped us to identify the temperamental and personality traits that were the best predictors of health, health behaviour, and QoL in the second measurement. The regression coefficients acquired in the final models are presented in Table 3.

**Table 3:** Temperamental and personality traits analyzed as predictors of health indicators in the second measurement.

Dependent variable Second measurement	Predictors First measurement	*β*	*t*	Δ*R*^2^	Value of *p*
Correct Eating Habits	Correct Eating Habits	0.68	6.16	0.457	0.001
Preventive Behavior	Preventive Behavior	0.44	3.49	0.262	0.001
	Sensory Sensitivity	0.27	2.09	0.067	0.042
Health Practices	Health Practices	0.44	3.30	0.206	0.002
Physical QoL	Physical QoL	0.56	4.21	0.312	0.001
BMI Index	BMI Index	0.89	10.78	0.789	0.001
Fat-free Mass	Fat-free Mass	0.95	23.04	0.931	0.001
	Perseveration	−0.08	−2.02	0.008	0.051
	Agreeableness	0.10	2.60	0.010	0.014
BMR	Basal Metabolic Rate	0.95	20.54	0.913	0.001
	Perseveration	−0.09	−2.07	0.010	0.046
	Agreeableness	0.12	2.67	0.014	0.012

*β, standardized regression coefficient; t, value of the test for predictors’ statistical significance; p, statistical significance; ΔR^2^, the change in determination coefficient*.

The values of each indicator from the first measurement correlated positively with the values from the second measurement. There was a positive relationship between Sensory Sensitivity and change in Preventive Behavior. The higher Sensory Sensitivity the greater the increase in Preventive Behavior. There were also negative relationships between Perseveration and the change in Fat-free Mass and BMR. Higher Perseveration was linked with smaller increase in Fat-free Mass and less reduction in BMR. Finally, there were positive relationships between Agreeableness and the change in Fat-free Mass and BMR. Higher Agreeableness was linked with greater increase in Fat-free Mass and greater BMR reduction. The temperamental and personality traits explained from 1.0 to 6.7% of the detected changes from the first measurement to the second measurement.

**Figure 1:**
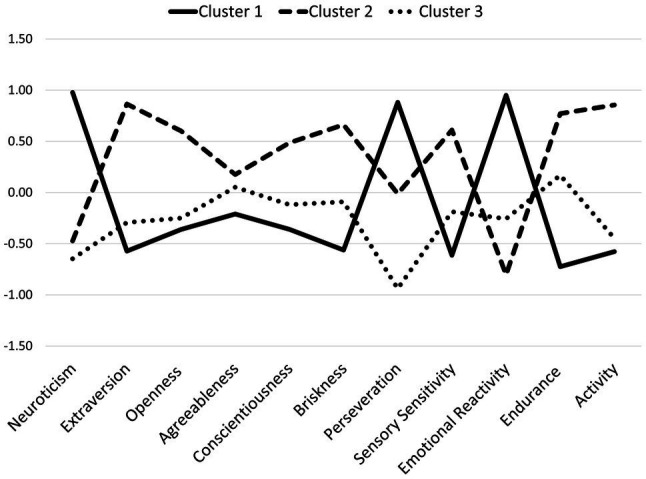
The final cluster centers in the three extracted types of temperamental and personality traits profiles. Cluster 1, higher Neuroticism, Perseveration, and Emotional Reactivity; Cluster 2, higher Extraversion, Briskness, Sensory Sensitivity, Endurance, Activity, and Conscientiousness; Cluster 3, lower Perseveration.

The next step in exploring the role of temperamental and personality traits was k-means cluster analysis. This analysis allows for the identification of subgroups of patients (i.e., clusters). All patients in a given subgroup thus identified have a similar profile of temperamental and personality traits. The profiles of patients from different subgroups have different profiles. This method tries to divide the subgroups such that they differ as much as possible. Three clusters were extracted. The final cluster centers are depicted in Figure 1.

There were statistically significant differences between the extracted clusters in terms of Neuroticism, *F (*2, 47)=27.84, *p*<0.001, *η*^2^=0.54, Extraversion, *F (*2, 47)=15.10, *p*<0.001, *η*^2^=0.39, Openness to Experience, *F (*2, 47)=5.26, *p*<0.01, *η*^2^=0.18, Conscientiousness, F (2, 47)=3.41, *p*<0.05, *η*^2^=0.13, Briskness, *F (*2, 47)=8.05, *p*<0.01, *η*^2^=0.26, Perseveration, *F (*2, 47)=24.83, *p*<0.001, *η*^2^=0.51, Sensory Sensitivity, *F (*2, 47)=8.72, *p*<0.01, *η*^2^=0.27, Emotional Reactivity, *F (*2, 47)=28.75, *p*<0.001, *η*^2^=0.55, Endurance, *F (*2, 47)=15.26, *p*<0.001 *η*^2^=0.39, and Activity, *F (*2, 47)=18.42, *p*<0.001 *η*^2^=0.44. The three clusters did not differ only in terms of Agreeableness, *F (*2, 47)=27.84, *p*>0.05. The patients from the first cluster (*n*=17) were characterized by higher Neuroticism, Perseveration, and Emotional Reactivity. The patients from the second cluster (*n*=16) had higher levels of Extraversion, Briskness, Sensory Sensitivity, Endurance, Activity, and Conscientiousness. The patients from the third cluster (*n*=17) had lower levels of Perseveration. Based on the results of the previous studies presented in the introduction to this paper (Lahmann et al., 2011; Gerlach et al., 2015; Garrido et al., 2018), it should be expected that the first cluster of traits would hinder adherence to the recommended guidelines, while the second cluster would foster behavioral changes and help to develop self-control. A lower level of Perseveration, which is the tendency to repeat emotional states even in the absence of the stimuli that evoked these states, does not have a clear, well-established impact on adherence to diet and physical activity guidelines.

In the next stage of statistical analysis, cluster membership was analyzed as a moderator of the changes between the two measurements, with the use of mixed-model repeated measure analysis of variance. The interaction effects between the cluster membership and the change from the first to the second measurement were tested in order to investigate moderation. Because it was possible that a change occurred only in a subgroup of participants, the analysis was performed for all of the dependent variables – not only for the variables for which a significant change was detected in the preliminary analysis presented in Table 2. Table 4 presents the values of the interaction effects tests.

**Table 4:** Statistical tests of interaction effects between cluster membership and the change from the first to the second measurement.

Variables	*F*	*η* ^2^	Value of *p*
Correct Eating Habits	1.19	0.05	0.314
Preventive Behavior	1.12	0.05	0.335
Health Practices	4.53	0.17	0.016
Positive Mental Attitude	0.11	0.01	0.896
Physical QoL	0.47	0.02	0.628
Emotional QoL	4.17	0.16	0.022
BMI Index	0.90	0.04	0.414
Fat Mass	3.18	0.13	0.051
Fat-free Mass	2.72	0.11	0.077
Intracellular Water	2.91	0.12	0.065
Basal Metabolic Rate	2.01	0.08	0.146

*F, interaction effect test value; p, statistical significance; η^2^, partial eta-squared*.

**Figure 2:**
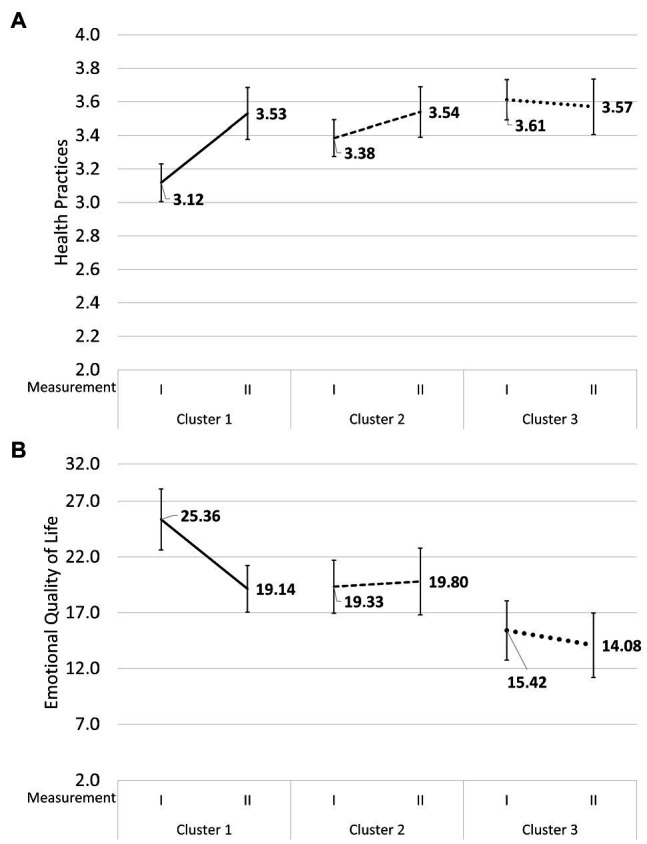
Mean values and 95% confidence intervals for Health Practices **(A)** and Emotional Quality of Life **(B)** at the first and the second measurements in the group of patients with higher Neuroticism, Perseveration, and Emotional Reactivity, in the group of patients with higher Extraversion, Briskness, Sensory Sensitivity, Endurance, and Activity, and in the group of patients with lower Perseveration.

Two interactions were statistically significant. The first one concerned Health Practices and the second one concerned Emotional Quality of Life. The level of Health Practices (i.e., daily habits pertaining to physical activity and rhythms of sleep and wakefulness) in the first measurement was significantly lower in the first cluster of patients with higher Neuroticism, Perseveration, and Emotional Reactivity, 95%CI: [3.01, 3.22], than in the second cluster of patients with higher Extraversion, Briskness, Sensory Sensitivity, Endurance, and Activity, 95%CI: [3.23, 3.53], or in the third cluster of patients, with lower Perseveration, 95%CI: [3.49, 3.73] (see Figure 2A). However, in the group of the patients from the first cluster, there was statistically significant progress: by the second measurement, they had reached the level of the patients from the second cluster, 95%CI: [3.38, 3.69]. The level of Health Practices in the second cluster, 95%CI: [3.39, 3.69], and in the third cluster, 95%CI: [3.41, 3.74], did not differ between the two consecutive measurements.

Similarly, the patients from the first cluster were characterized by lower Emotional Quality of Life at the first measurement, 95%CI: [22.69, 28.01], but progressed significantly, 95%CI: [17.01, 21.18], and at the second measurement had the same levels as the patients from the second cluster, 95%CI: [16.99, 21.79] and the third cluster, 95%CI: [12.88, 18.02] (in the Polish version of the SF-36, higher scores mean lower QoL while lower scores mean a higher QoL), while the patients from the second cluster, 95%CI: [16.84, 22.83], and the third cluster, 95%CI: [11.17, 16.97], were at the same level at both measurements (see Figure 2B).

The level of Health Practices at the second measurement correlated negatively with the indicator of Emotional Quality of Life in the total sample, *r* (48)=−0.26, *p*<0.05. Moreover, only in the group of the patients from the second cluster, with higher Extraversion, Briskness, Sensory Sensitivity, Endurance, and Activity, did the level of Health Practices at the first measurement correlate negatively with the indicator of Emotional Quality of Life at the second measurement, *r* (15)=−0.44, *p*<0.05, and the indicator of Emotional Quality of Life at the first measurement correlate negatively with the level of Health Practices at the second measurement, *r* (15)=−0.47, *p*<0.05.

## Discussion

All patients improved over the course of the 15months in terms of Physical Quality of Life, Eating Habits, adherence to doctors’ advice and treatment, rhythms of sleep and wakefulness, and physical activity. The improvement could also be seen in the lower BMI values, lower BMR values, and the higher values of Fat-free Mass. Hypothesis 1 was confirmed by the results. Opportunities for improvement in groups of patients diagnosed with metabolic syndrome have also been demonstrated in other studies (Lin et al., 2014; Barnes and Barber, 2017), even when the psychological interventions had a very limited scope – for example, when they were home-based (Blackford et al., 2016) or carried out by phone (Lin et al., 2016). We found that higher Sensory Sensitivity, lower Perseveration, and higher Agreeableness foster positive change. In the group of patients with higher Neuroticism, Perseveration, and Emotional Reactivity, levels of Emotional Quality of Life and Health Practices were significantly lower in comparison with the patients with higher Extraversion, Endurance, and Activity and in comparison with the patients with lower Perseveration. Lower levels of Health Practices were accompanied by lower levels of Emotional Quality of Life in the whole sample. As expected, patients with higher Neuroticism, Perseveration, and Emotional Reactivity were in worse condition in both of these areas. These three characteristics make engaging in compulsive eating behaviors in order to discharge psychological tension more likely. Neuroticism has been demonstrated to be associated with lower QoL (Bobić, 2012). Perseveration and Emotional Reactivity were also found to be negatively associated with QoL, for example, in patients diagnosed with malignant neoplasm (Burnos and Bargiel-Matusiewicz, 2018). However, the patients with all three adverse factors (i.e., those with higher Neuroticism, Perseveration, and Emotional Reactivity) improved in terms of Emotional Quality of Life and Health Practices, attaining the same level as other patients, while patients with other profiles of temperamental and personality traits did not change, which supports hypothesis 2. Higher levels of Neuroticism, Perseveration, and Emotional Reactivity can lead to an adverse pattern of emotional regulation, characterized by internalizing psychological tension instead of efficient coping, *inter alia*, through physical activity (Robinson et al., 2006; Boyes et al., 2017). The persistent ruminations and worry associated with Neuroticism can also lead to somatic complaints (Denovan et al., 2019). This process can also have an adverse effect on quality of sleep by dysregulating rhythms of sleep and wakefulness. Daily rumination and negative affect have been found to be mediators of the relationship between Neuroticism and impaired sleep (Slavish et al., 2018). Emotional Quality of Life can be affected both directly by ineffective stress management and indirectly through poor sleep quality. Poor Emotional Quality of Life can, in turn, negatively affect coping mechanisms by instigating a sort of vicious circle of maladaptive behaviors. However, the mechanisms and interrelations between Emotional Quality of Life and stress management still need further investigation through longitudinal studies (Gutiérrez Dona, 2003). The results of this study suggest that an individual motivational approach can be effective in this group of patients. They achieved parity in terms of Emotional Quality of Life and Health Practices with other patients and also improved their rhythms of sleep and wakefulness and enhanced their physical activity, which are accompanied by better Emotional Quality of Life. It seems that motivational counseling is effective for patients diagnosed with metabolic syndrome in general, but patients with higher Neuroticism, Perseveration, and Emotional Reactivity can benefit even more, as they are often in poorer psychological condition before the motivational intervention. For them, individual motivational intervention may also be particularly useful, because patients with higher Neuroticism are known to have higher dropout rates from periodic health screenings (Armon and Toker, 2013).

The relationship between Health Practices and Emotional Quality of Life is best seen in the group of patients with higher Extraversion, Briskness, Sensory Sensitivity, Endurance, and Activity, who also display a straightforward link between emotional states and task-oriented coping (Kaiseler et al., 2019). The pattern by which Emotional Quality is related to Health Practices measured after 15months and Emotional Quality reflects Health Practices 15months ago suggests a reciprocal relationship.

Personality and temperamental traits explain, to some extent, the functioning of patients with metabolic syndrome in the area of emotional regulation. However, even the patients with the most disadvantageous temperamental personality profile can undergo positive change to at least equal other patients in terms of Health Practices and Emotional Quality of Life. The scores acquired in the second measurement in this group of patients differed from the other patients by less than 5%.

The current study has certain limitations. Firstly, there were only two measurements. The conclusions would be stronger if they were based on a longer period and/or on a greater number of measurements. It would also make plotting the trajectories of changes possible. Secondly, cluster analysis allows many aspects of patients’ functioning to be taken into consideration, but it also imposes the existence of clusters. The characteristics of each extracted subgroup are purely empirical. However, the algorithm does not assure their replicability. This means that the identified clusters of patients may differ in subsequent studies.

Furthermore, the conclusions from this study can be generalized only to patients with all properties of the profiles. For example, in this study, we describe a group of patients with higher Neuroticism, Perseveration, and Emotional Reactivity. The conclusions drawn relate to such patients, but not to patients who only have higher Neuroticism without elevated Perseveration and Emotional Reactivity. The current study was limited to only a clinical group, with no control group. A control group without any motivational intervention could strengthen the conclusions drawn. Future research should examine a variety of motivational interventions on a larger sample. This would allow researchers to examine, for example, whether exploring the long-term health consequences of metabolic syndrome or addressing patients’ ambivalence and uncertainty are necessary elements of effective motivational interventions.

## Data Availability Statement

The raw data supporting the conclusions of this article will be made available by the authors, without undue reservation.

## Ethics Statement

The studies involving human participants were reviewed and approved by the Bioethical Committee of the Military Institute of Medicine. The patients/participants provided their written informed consent to participate in this study.

## Author Contributions

AB and AS contributed to conception and design of the study, and wrote sections of the manuscript. AB organized the database, performed the statistical analysis, and wrote the first draft of the manuscript. All authors contributed to manuscript revision, read, and approved the submitted version.

## Funding

The research was funded by subvention of the Ministry of Education and Science. The publication was supported by the Faculty of Psychology, University of Warsaw, from the funds awarded by the Ministry of Science and Higher Education in the form of a subsidy for the maintenance and development of research potential in 2021 (501-D125-01-1250000 zlec*.5011000229).

## Conflict of Interest

The authors declare that the research was conducted in the absence of any commercial or financial relationships that could be construed as a potential conflict of interest.

## Publisher’s Note

All claims expressed in this article are solely those of the authors and do not necessarily represent those of their affiliated organizations, or those of the publisher, the editors and the reviewers. Any product that may be evaluated in this article, or claim that may be made by its manufacturer, is not guaranteed or endorsed by the publisher.

## References

[ref1] Alberti K. G. Zimmet P. Z. (1998). Definition, diagnosis and classification of diabetes mellitus and its complications. Part 1: diagnosis and classification of diabetes mellitus provisional report of a WHO consultation. Diabet. Med. 15, 539–553. doi: 10.1002/(SICI)1096-9136(199807)15:7<539::AID-DIA668>3.0.CO;2-S, PMID: 9686693

[ref2] Armon G. Toker S. (2013). The role of personality in predicting repeat participation in periodic health screening. J. Pers. 81, 452–464. doi: 10.1111/jopy.12021, PMID: 23126563

[ref3] Barnes R. D. Barber J. A. (2017). Preliminary examination of metabolic syndrome response to motivational interviewing for weight loss as compared to an attentional control and usual care in primary care for individuals with and without binge-eating disorder. Eat. Behav. 26, 108–113. doi: 10.1016/j.eatbeh.2017.02.007, PMID: 28226308 PMC5545172

[ref4] Barrès R. Zierath J. R. (2016). The role of diet and exercise in the transgenerational epigenetic landscape of T2DM. Nat. Rev. Endocrinol. 12, 441–451. doi: 10.1038/nrendo.2016.87, PMID: 27312865

[ref5] Bird S. R. Hawley J. A. (2017). Update on the effects of physical activity on insulin sensitivity in humans. BMJ Open Sport Exerc. Med. 2:e000143. doi: 10.1136/bmjsem-2016-000143, PMID: 28879026 PMC5569266

[ref6] Blackford K. Jancey J. Lee A. H. James A. Howat P. Waddell T. (2016). Effects of a home-based intervention on diet and physical activity behaviors for rural adults with or at risk of metabolic syndrome: a randomised controlled trial. Int. J. Behav. Nutr. Phys. Act. 13, 1–10. doi: 10.1186/s12966-016-0337-2 26830197 PMC4736250

[ref7] Bobić J. (2012). Subjective estimation of the quality of life in relation to neuroticism. Arh. Hig. Rada Toksikol. 63, 17–22. doi: 10.2478/10004-1254-63-2012-2141, PMID: 22548849

[ref8] Boyes M. E. Carmody T. M. Clarke P. J. F. Hasking P. A. (2017). Emotional reactivity and perseveration: independent dimensions of trait positive and negative affectivity and differential associations with psychological distress. Personal. Individ. Differ. 105, 70–77. doi: 10.1016/j.paid.2016.09.025

[ref9] Burnos A. Bargiel-Matusiewicz K. M. (2018). Quality of life and PTSD symptoms, and temperament and coping With stress. Front. Psychol. 9:2072. doi: 10.3389/fpsyg.2018.02072, PMID: 30443229 PMC6221927

[ref10] Clark R. L. Infante A. M. Cuff C. F. Mark Olfert I. Holásková I. McFadden J. W.. (2019). Educational intervention improves fruit and vegetable intake in young adults with metabolic syndrome components. Nutr. Res. 62, 89–100. doi: 10.1016/j.nutres.2018.11.010, PMID: 30803510 PMC6392018

[ref11] Cloninger C. R. Cloninger K. M. Zwir I. Keltikangas-Järvinen L. (2019). The complex genetics and biology of human temperament: a review of traditional concepts in relation to new molecular findings. Transl. Psychiatry 9:290. doi: 10.1038/s41398-019-0621-4, PMID: 31712636 PMC6848211

[ref12] de la Iglesia R. Lopez-Legarrea P. Abete I. Bondia-Pons I. Navas-Carretero S. Forga L.. (2014). A new dietary strategy for long-term treatment of the metabolic syndrome is compared with the American Heart Association (AHA) guidelines: the MEtabolic syndrome REduction in NAvarra (RESMENA) project. Br. J. Nutr. 111, 643–652. doi: 10.1017/S0007114513002778, PMID: 23968597

[ref13] Denovan A. Dagnall N. Lofthouse G. (2019). Neuroticism and somatic complaints: concomitant effects of rumination and worry. Behav. Cogn. Psychother. 47, 431–445. doi: 10.1017/S1352465818000619, PMID: 30400997

[ref14] Dohnke B. Nowossadeck E. Müller-Fahrnow W. (2010). Motivation and participation in a phase III cardiac rehabilitation programme: an application of the health action process approach. Res. Sports Med. 18, 219–235. doi: 10.1080/15438627.2010.510032, PMID: 21058208

[ref15] Dudek D. Siwek M. Jaeschke R. Dembińska-Kieć A. Arciszewska A. Hebal F.. (2015). Relationships between obesity, bipolar spectrum features, and personality traits: a case-control study. Eur. Rev. Med. Pharmacol. Sci. 19, 4235–4240. PMID: 26636508

[ref16] Garrido S. J. Funes P. N. Peñaloza Merlo M. E. Cupani M. (2018). Personality traits associated with eating disorders and obesity in young Argentineans. Eat. Weight Disord. 23, 571–579. doi: 10.1007/s40519-018-0546-6, PMID: 30043159

[ref17] Gerlach G. Herpertz S. Loeber S. (2015). Personality traits and obesity: a systematic review (PSYNDEXshort). Obes. Rev. 16, 32–63. doi: 10.1111/obr.12235, PMID: 25470329

[ref18] Gochman D. S. (1988). Health Behavior. New York: Plenum Press.

[ref19] Gutiérrez Dona E. B. (2003). Coping with stress at work: a longitudinal study on health outcomes and quality of life. dissertation/master’s thesis.

[ref20] Hosseini Z. Veenstra G. Khan N. A. Conklin A. I. (2020). Associations between social connections, their interactions, and obesity differ by gender: a population-based, cross-sectional analysis of the Canadian longitudinal study on aging. PLoS One 15:e0235977. doi: 10.1371/journal.pone.0235977, PMID: 32730260 PMC7392536

[ref21] Juczyński Z. (2012). Measurement Tools in Health Promotion and Psychology. Warsaw: Psychological Test Laboratory of the Polish Psychological Association.

[ref22] Kaiseler M. Levy A. Nicholls A. R. Madigan D. J. (2019). The independent and interactive effects of the big-five personality dimensions upon dispositional coping and coping effectiveness in sport. Int. J. Sport Exercise Psychol. 17, 410–426. doi: 10.1080/1612197X.2017.1362459

[ref23] Kalinowski P. Mianowana M. (2016). Metabolic syndrome part II: epidemiology of metabolic syndrome in Poland and in the world. J. Educ. Health Sport 6, 466–480. doi: 10.5281/zenodo.50681

[ref24] Kuo C.-C. Lin C.-C. Tsai F.-M. (2014). Effectiveness of empowerment-based self-management interventions on patients with chronic metabolic diseases: a systematic review and meta-analysis. Worldviews Evid.-Based Nurs. 11, 301–315. doi: 10.1111/wvn.12066, PMID: 25327253

[ref25] Lahmann C. Henrich G. Henningsen P. Baessler A. Fischer M. Loew. (2011). The impact of personality traits on the success of a multimodal obesity treatment. Behav. Med. 37, 119–124. doi: 10.1080/08964289.2011.635169, PMID: 22168328

[ref26] Lin C.-H. Chiang S.-L. Heitkemper M. M. Hung Y.-J. Lee M.-S. Tzeng W.-C.. (2016). Effects of telephone-based motivational interviewing in lifestyle modification program on reducing metabolic risks in middle-aged and older women with metabolic syndrome: a randomized controlled trial. Int. J. Nurs. Stud. 60, 12–23. doi: 10.1016/j.ijnurstu.2016.03.003, PMID: 27297365

[ref27] Lin C.-H. Chiang S.-L. Tzeng W.-C. Chiang L.-C. (2014). Systematic review of impact of lifestyle-modification programs on metabolic risks and patient-reported outcomes in adults with metabolic syndrome. Worldviews Evid.-Based Nurs. 11, 361–368. doi: 10.1111/wvn.12069, PMID: 25488565

[ref28] Locke A. E. Kahali B. Berndt S. I. Justice A. E. Pers T. H. Day F. R.. (2015). Genetic studies of body mass index yield new insights for obesity biology. Nature 518, 197–206. doi: 10.1038/nature14177, PMID: 25673413 PMC4382211

[ref29] McCrae R. R. Costa P. T. (1999). A Five-Factor Theory of Personality. New York: Guilford Press.

[ref30] McGrane N. Galvin R. Cusack T. Stokes E. (2015). Addition of motivational interventions to exercise and traditional physiotherapy: a review and meta-analysis. Physiotherapy 101, 1–12. doi: 10.1016/j.physio.2014.04.009, PMID: 25239472

[ref31] Miller W. R. Rose G. R. (2009). Toward a theory of motivational interviewing. Am. Psychol. 64, 527–537. doi: 10.1037/a0016830, PMID: 19739882 PMC2759607

[ref32] Mommersteeg P. M. C. Pouwer F. (2012). Personality as a risk factor for the metabolic syndrome: a systematic review. J. Psychosom. Res. 73, 326–333. doi: 10.1016/j.jpsychores.2012.08.019, PMID: 23062804

[ref33] Oldridge N. B. Stoedefalke K. G. (1984). Compliance and motivation in cardiac exercise programs. Clin. Sports Med. 3, 443–454. doi: 10.1016/S0278-5919(20)31338-7, PMID: 6388859

[ref34] Olson E. A. Mullen S. P. Raine L. B. Kramer A. F. Hillman C. H. McAuley E. (2017). Integrated social- and neurocognitive model of physical activity behavior in older adults with metabolic disease. Ann. Behav. Med. 51, 272–281. doi: 10.1007/s12160-016-9850-4, PMID: 27844326 PMC5475366

[ref35] Oniszczenko W. Dragan W. Chmura A. Lisik W. (2015). Temperament as a risk factor for obesity and affective disorders in obese patients in a polish sample. Eat. Weight Disord. 20, 233–239. doi: 10.1007/s40519-014-0151-2, PMID: 25155162

[ref36] Peeters A. Backholer K. (2017). How to influence the obesity landscape using health policies. Int. J. Obesity 41, 835–839. doi: 10.1038/ijo.2017.24, PMID: 28127043

[ref37] Reshadat S. Zakiei A. Hatamin P. Bagheri A. Rostami S. Komasi S. (2017). A study of the correlation of personality traits (neuroticism and psychoticism) and self-efficacy in weight control with unhealthy eating behaviors and attitudes. Ann. Med. Health Sci. Res. 7, 32–38.

[ref38] Robinson M. D. Wilkowski B. M. Kirkeby B. S. Meier B. P. (2006). Stuck in a rut: perseverative response tendencies and the neuroticism-distress relationship. J. Exp. Psychol. Gen. 135, 78–91. doi: 10.1037/0096-3445.135.1.78, PMID: 16478317

[ref39] Saklayen M. G. (2018). The global epidemic of the metabolic syndrome. Curr. Hypertens. Rep. 20:12. doi: 10.1007/s11906-018-0812-z, PMID: 29480368 PMC5866840

[ref40] Salas-Salvadó J. Bulló M. Estruch R. Ros E. Covas M.-I. Ibarrola-Jurado N.. (2014). Prevention of diabetes with Mediterranean diets: a subgroup analysis of a randomized trial. Ann. Intern. Med. 160, 1–10. doi: 10.7326/M13-1725, PMID: 24573661

[ref41] Slavish D. C. Sliwinski M. J. Smyth J. M. Almeida D. M. Lipton R. B. Katz M. J.. (2018). Neuroticism, rumination, negative affect, and sleep: examining between- and within-person associations. Personal. Individ. Differ. 123, 217–222. doi: 10.1016/j.paid.2017.11.023, PMID: 29610545 PMC5877474

[ref42] Stanyte A. Smigelskas K. (2018). Personality traits and problematic eating behavior as predictors of dietary habits in young adults. Age 21, 2–3.

[ref43] Strelau J. (1996). The regulative theory of temperament: current status. Personal. Individ. Differ. 20, 131–142. doi: 10.1016/0191-8869(95)00159-X

[ref44] Strelau J. (2006). Temperament Jako Regulator Zachowania z Perspektywy półwiecza badań [Temperament as the Regulator of Behavior from the Perspective of half a Century Research]. Gdańsk: Gdańskie Wydawnictwo Psychologiczne.

[ref45] Strelau J. Zawadzki B. (1995). The formal characteristics of behavior-temperament inventory (FCB-TI): validity studies. Eur. J. Personal. 9, 207–229. doi: 10.1002/per.2410090304

[ref46] Susin N. de Melo Boff R. Ludwig M. W. B. Feoli A. M. P. da Silva A. G. Macagnan F. E.. (2016). Predictors of adherence in a prevention program for patients with metabolic syndrome. J. Health Psychol. 21, 2156–2167. doi: 10.1177/1359105315572451, PMID: 25805660

[ref47] Sutin A. R. Costa P. T. Jr. Uda M. Ferrucci L. Schlessinger D. Terracciano A. (2010). Personality and metabolic syndrome. Age 32, 513–519. doi: 10.1007/s11357-010-9153-9, PMID: 20567927 PMC2980597

[ref48] Sutin A. R. Terracciano A. (2016). Personality traits and body mass index: modifiers and mechanisms. Psychol. Health 31, 259–275. doi: 10.1080/08870446.2015.1082561, PMID: 26274568 PMC4827155

[ref49] Sutin A. R. Terracciano A. (2018). Mother and child personality traits associated with common feeding strategies and child body mass index. Appetite 125, 295–301. doi: 10.1016/j.appet.2018.02.009, PMID: 29454016 PMC6289273

[ref50] Terracciano A. Schrack J. A. Sutin A. R. Chan W. Simonsick E. M. Ferrucci L. (2013). Personality, metabolic rate and aerobic capacity. PLoS One 8:e54746. doi: 10.1371/journal.pone.0054746, PMID: 23372763 PMC3556088

[ref51] The World Health Organization Quality of Life assessment (WHOQOL) (1995). Position paper from the World Health Organization. Soc. Sci. Med. 41, 1403–1409. doi: 10.1016/0277-9536(95)00112-K, PMID: 8560308

[ref52] Tylka J. Piotrowicz R. (2009). Quality of life measure SF-36 – polish version. Kardiol. Pol. 67, 1166–1169. PMID: 20209678

[ref53] Walther M. Hilbert A. (2016). Temperament dispositions, problematic eating behaviors and overweight in adolescents. Eur. Eat. Disord. Rev. 24, 19–25. doi: 10.1002/erv.2381, PMID: 26104832

[ref54] Zawadzki Z. Strelau J. Szczepaniak P. Śliwińska M. (1998). NEO-FFI. Personality Inventory. Warsaw: Psychological Test Laboratory of the Polish Psychological Association.

